# Addressing opioid tolerance and opioid‐induced hypersensitivity: Recent developments and future therapeutic strategies

**DOI:** 10.1002/prp2.789

**Published:** 2021-06-06

**Authors:** Faris Khan, Aman Mehan

**Affiliations:** ^1^ School of Clinical Medicine University of Cambridge Cambridge UK

**Keywords:** analgesia, opioids, opioid‐induced hypersensitivity, opioid receptors, opioid tolerance, pain

## Abstract

Opioids are a commonly prescribed and efficacious medication for the treatment of chronic pain but major side effects such as addiction, respiratory depression, analgesic tolerance, and paradoxical pain hypersensitivity make them inadequate and unsafe for patients requiring long‐term pain management. This review summarizes recent advances in our understanding of the outcomes of chronic opioid administration to lay the foundation for the development of novel pharmacological strategies that attenuate opioid tolerance and hypersensitivity; the two main physiological mechanisms underlying the inadequacies of current therapeutic strategies. We also explore mechanistic similarities between the development of neuropathic pain states, opioid tolerance, and hypersensitivity which may explain opioids’ lack of efficacy in certain patients. The findings challenge the current direction of analgesic research in developing non‐opioid alternatives and we suggest that improving opioids, rather than replacing them, will be a fruitful avenue for future research.

## Graphical abstract

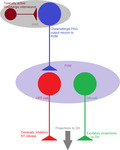

**Abbreviations**
DH: dorsal horn
DOR: delta
GPCRs: G‐protein‐coupled receptors
KOR: kappa
MOR: the mu
OIH: opioid‐induced hypersensitivity

## 1 INTRODUCTION

Pain is “*an unpleasant sensory and emotional experience associated with actual or potential tissue damage, or described in terms of such damage*.”^1^ Pain, and specifically its treatment, is a major issue facing healthcare systems globally. In the US, the average annual cost associated with pain is $600 billion.^2^ Chronic pain is undertreated in 80% of cases.^3^ Opioids are the most effective compounds for alleviating severe pain across a wide range of conditions, for example, acute pain in response to injury or chronic pain in response to inflammatory disease or cancer. However, opioid use is associated with side effects that become more severe with increased use, for example, severe drowsiness and breathing difficulties. Furthermore, decreasing analgesic efficacy is observed clinically with prolonged use of any strong opioid; this is a combination of tolerance to their analgesic effect and the development of opioid‐induced hypersensitivity (OIH) within pain signaling pathways. Tolerance is defined as a decreased efficacy following repeated administration,^4^ whereas OIH is a state of increased pain sensitivity.^5^ These can be overcome by increased dosage; however, this increases the risk of more severe side effects (e.g., respiratory depression). Thus, a vicious circle exists, and patients are often faced with a choice between side effects or inadequate analgesia. In addition, opioid‐tolerant patients require significantly longer lengths of stay in hospital and have higher readmission rates compared with patients not on opioids,^6^ contributing to opioids’ economic burden. Therefore, there is an unmet clinical need to address tolerance.

Non‐opioid painkillers currently available either do not offer sufficient analgesia (e.g., non‐steroidal anti‐inflammatory drugs [NSAIDs]) or are only efficacious in specific pain condition (e.g., gabapentinoids in neuropathic pain), and are also limited by their own adverse side‐effects (e.g., gastrointestinal bleeding with NSAIDs). An alternative approach is improving opioids by reducing the development of opioid tolerance and OIH, increasing their long‐term efficacy.

This review will explore the proposed mechanisms of OIH and tolerance and identify areas of therapeutic potential that may improve the use of currently available opioids. Furthermore, it will discuss mechanistic similarities between the development of opioid tolerance/OIH and certain neuropathic pain conditions which may provide an explanation for opioids’ lack of efficacy in some conditions.

## 2 OPIOID SIGNALING

The analgesic properties of opium, an extract from the *Papaver somniferum* poppy, and from which modern opiates (such as morphine) are derived, have been recognized for centuries. This beneficial effect is offset by a variety of side‐effects including constipation, respiratory depression, dependence, addiction, hypersensitivity, and tolerance.

Opioids exert their action through interaction with the superfamily of heterotrimeric opioid G‐protein‐coupled receptors (GPCRs); the mu (MOR), delta (DOR), and kappa (KOR) opioid receptors. The most clinically relevant are MORs; it is through these receptors that both natural opiates and synthetic opioids, such as fentanyl, exert analgesia and their side‐effects. This is confirmed by numerous studies using MOR‐knockout mice which no longer respond to morphine to produce analgesia or its side‐effects.^7^ In contrast, DOR‐knockouts retain full morphine analgesia,^8^ and more recent studies have suggested that while activation of KOR can cause analgesia, extremely high opioid doses were required for mild analgesia, and further increase in dosage was not possible due to the paradoxical pain hypersensitivity observed.^9^

Attempting to improve opioid analgesia is not a novel idea. Strategies to reduce side‐effects associated with prolonged opioid use, such as the use of KOR and DOR agonists or peripheral MOR antagonists, have been trialed but with limited success. DOR and KOR agonists produce limited analgesia in only specific inflammatory conditions, and KOR agonists may be specific for visceral pain.^10^, ^11^, ^12^ Also, KOR agonists have distinct central nervous system (CNS) side‐effects, such as dysphoria, sedation, and psychosis, when compared to those traditionally associated with opioids.^13^ Peripheral MOR antagonism acts on the gastrointestinal (GI) system, only reducing constipation and preserving centrally mediated side‐effects.^14^ This highlights an unmet clinical need to reduce the critical CNS‐mediated side effects of opioids (tolerance and OIH).

## 3 PAIN SIGNALING PATHWAYS

Pain processing is initiated by the activation of nociceptive afferent neurons (C and Aδ fibers). The afferents terminate in the dorsal horn (DH) of the spinal cord, where they synapse with second‐order neurons, either projecting neurons that carry the signal to supraspinal regions, or interneurons within the DH which later synapse onto projecting neurons. This synapse is the first possible point of regulation of pain signaling, occurring through inhibitory interneurons and descending control. Regulation is through inhibitory neurotransmitters (NTs) such as gamma‐aminobutyric acid (GABA) or endogenous opioids which create a “gate” for incoming pain signals.^15^ Therefore, opioid receptors are present on primary afferent and in the DH, and local administration of opioids into the spinal cord induces analgesia.^16^

**FIGURE 1:**
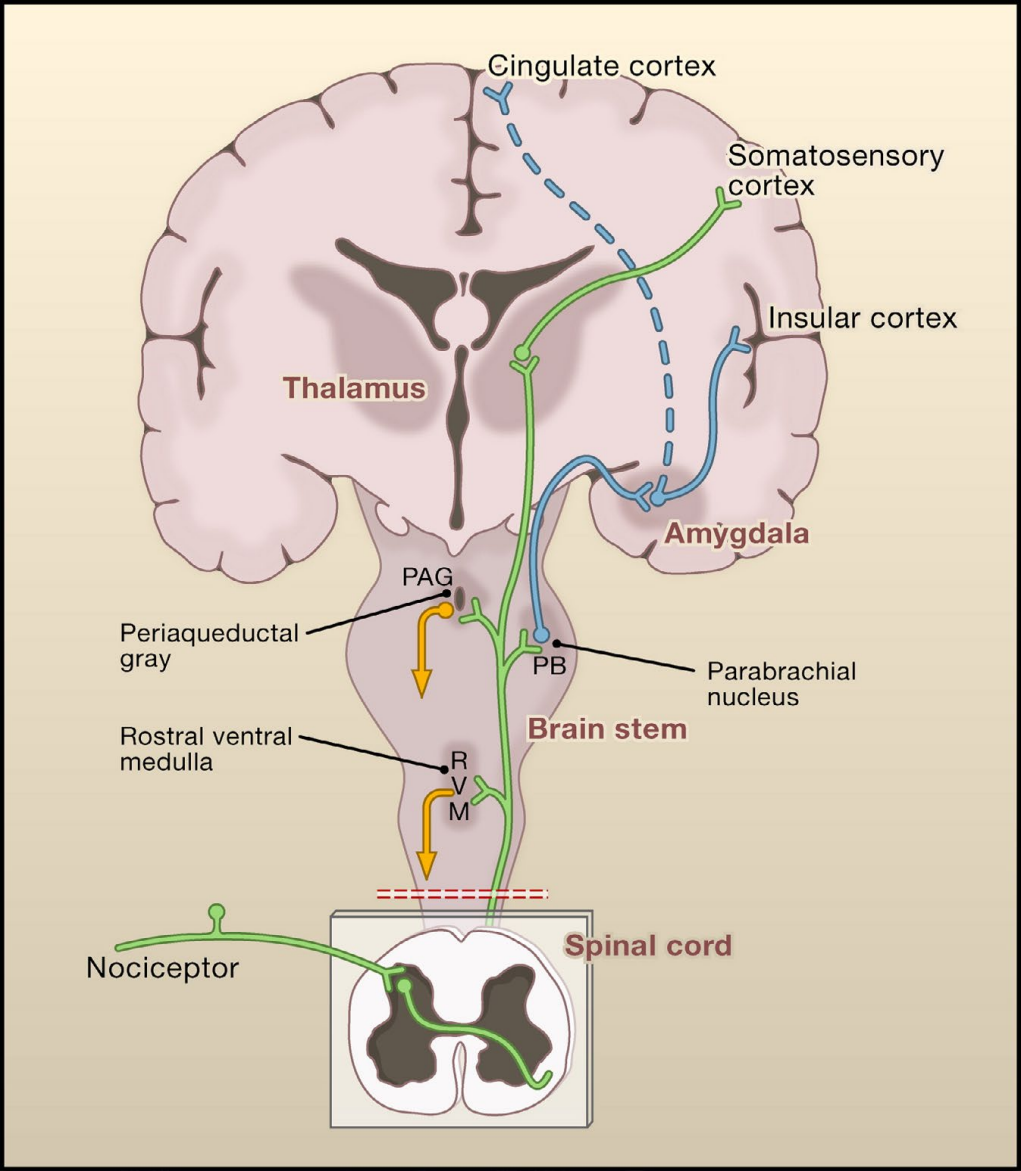
Anatomy of the pain processing pathway (From Cellular and Molecular Mechanisms of Pain, Basbaum et al., Cell, 2009, 139:267–284. With permission from Elsevier).^17^ Primary afferent nociceptors convey noxious information to projecting neurons in the dorsal horn (DH) of the spinal cord. A subset of these projecting neurons transmits information to the somatosensory cortex via the thalamus providing physical information about the painful stimulus (the primary ascending pathway, *in green*). Other projection neurons (*in blue*) relay via brainstem structures to engage the insular and cingulate cortex, contributing to the affective and cognitive components of pain. The ascending information is also able to interact with several other brain/brainstem areas, such as the rostral ventral medulla (RVM) and midbrain periaqueductal gray (PAG) to engage descending feedback systems that regulate the output of projecting neurons in the DH (*in orange*)

Projection neurons exit the DH toward supraspinal regions important for the conscious sensation of pain. For example, the somatosensory cortex for localization and intensity and the cingulate cortex, insula, and amygdala for the cognitive and emotional components associated with painful stimuli. This ascending system also makes a crucial connection with regions of the midbrain and brainstem that feed into the descending inhibitory pathways (Figure 1).

**FIGURE 2:**
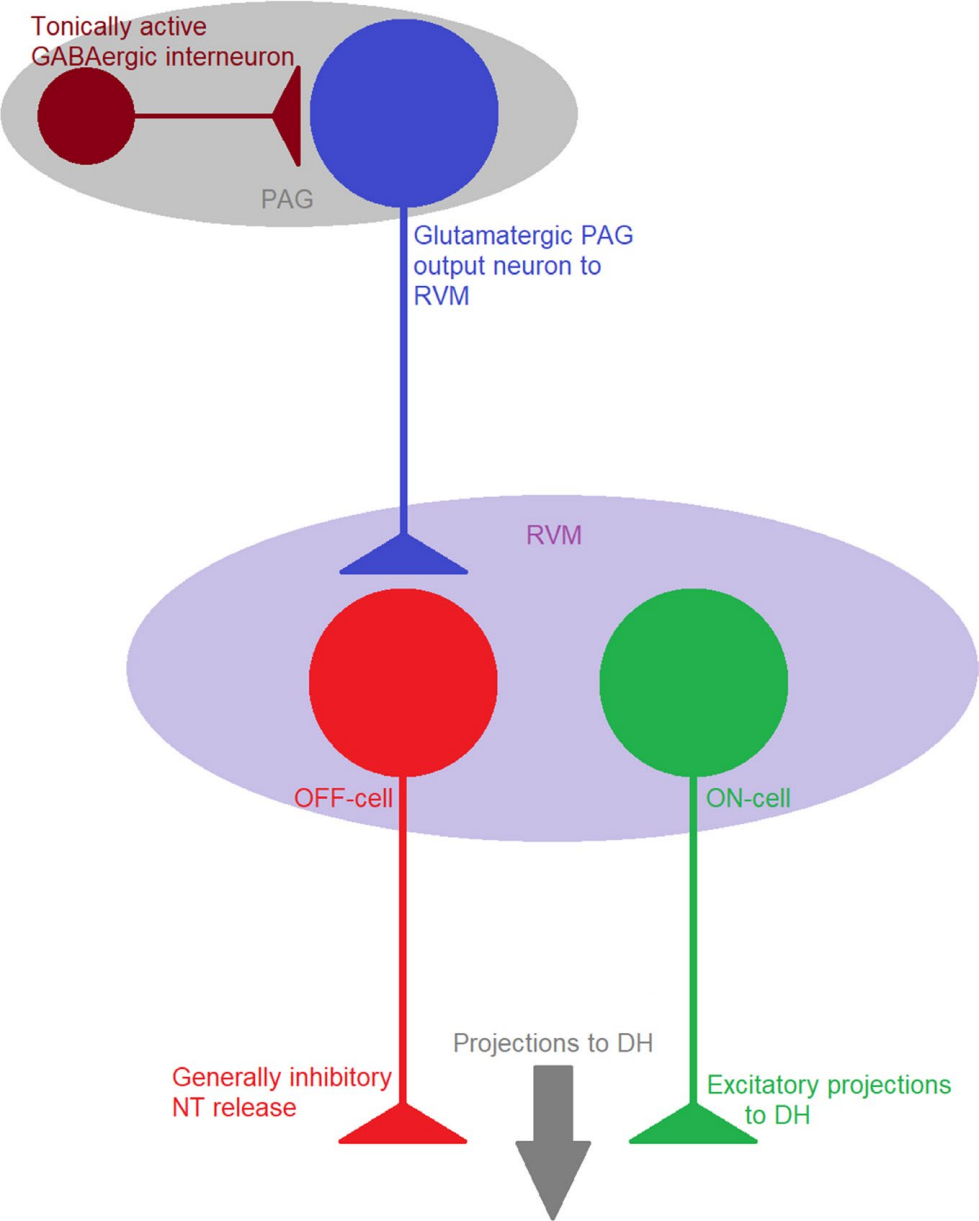
The PAG‐RVM descending system under normal conditions. In the naïve state, GABAergic interneurons are tonically active, thus both PAG output neurons and OFF‐cells have low spontaneous firing rates. Activity in OFF‐cells causes antinociception, and activity in ON‐cells represents descending facilitation of pain. The normal activity of ON‐cells is also low, such that the overall balance between ON‐cell and OFF‐cell output to the dorsal horn (DH) is equal and there is no net hypersensitive or antinociceptive state in the individual

The descending pain pathways are primarily antinociceptive, the most studied being the periaqueductal gray‐rostroventral medulla‐dorsal horn (PAG‐RVM‐DH) circuit (Figure 2), but activation of certain supraspinal regions (e.g., the anterior cingulate cortex) can also give rise to descending facilitation which can counteract descending inhibition from the PAG and RVM, or contribute to pain hypersensitivity in certain injury states.^18^, ^19^ The PAG may also be involved in some forms of descending facilitation and two classes of nociceptive modulating neurons have been noted in this region.^20^

The RVM is the central hub for the descending systems (both facilitatory and inhibitory). Three populations of neurons projecting to the spinal cord from the RVM have been identified from animal electrophysiological experiments (ON‐cells, OFF‐cells, and neutral cells) on the basis of their pronociceptive/antinociceptive effects following stimulation.^21^ Prior to a tail‐flick reflex, ON‐cells show a burst in activity while the firing rate of OFF‐cells is dramatically reduced.^22^ Signaling of painful stimuli is associated with increased activity in ON‐cells, necessary for the acute hypersensitivity observed in animals, and suppression of antinociceptive OFF‐cells.^23^ Opioid receptors are also located throughout the PAG‐RVM‐DH descending system and can be activated endogenously to induce analgesia, for example, during the phenomena of stress‐induced analgesia.^24^ Therefore, opioid receptors are expressed consistently in the pain processing pathways (ascending and descending), perhaps explaining their high efficacy as analgesic targets.

## 4 SITES OF OPIOID‐INDUCED ANALGESIA

There is compelling evidence suggesting the supraspinal action of exogenous opioids is primarily responsible for their antinociceptive effect. Direct administration of the MOR antagonist, naloxone, into the ventrolateral PAG (vlPAG) blocks the antinociceptive effect of systemically administered morphine,^25^ and administration of a MOR agonist into the vlPAG produces analgesia in rats that is blocked by systemically administered naloxone.^26^ These data suggest the vlPAG is an essential site for exogenous opioid‐mediated analgesia.

The mechanism of action in the PAG involves disinhibition of PAG output neurons to OFF‐cells in the RVM, via inhibition of GABA release from GABAergic interneurons. Under normal conditions, these interneurons have tonic activity. Upon agonist binding to MORs, the activity of these neurons is decreased.^27^, ^28^ Microdialysis of the PAG following acute morphine administration showed decreased GABA levels.^29^ More recent studies using chemogenetic manipulation of vlPAG neural activity provide further support for this crucial mechanism toward opioid‐induced analgesia.^30^ The selective inhibition of GABAergic neurons or selectively activating glutamatergic (output) neurons in the vlPAG mimicked the antinociceptive effects of opioids.

The OFF‐cell population of RVM neurons appears to primarily consist of Glycine/GABAergic neurons that act diffusely in the dorsal horn to decrease excitability.^31^, ^32^ Some OFF‐cells may provide glutamatergic projections to endogenous opioid/GABA releasing inhibitory interneurons in the dorsal horn^33^ to provide a mechanism for precise inhibition of specific pain inputs.

Furthermore, microinjection of MOR agonists into the RVM directly inhibits ON‐cells.^34^, ^35^ This combination of indirect disinhibition of OFF‐cells and direct inhibition of ON‐cells projecting to the dorsal horn appears to be the primary mechanism of opioid analgesia. Khalefa et al. attempted to quantify the relative contributions of peripheral, spinal, and supraspinal MORs to the analgesic effects of systemic opioids in a rat model of inflammatory pain.^16^ In agreement with the previous experiments discussed, antagonism of the supraspinal effects of fentanyl and morphine with intracerebrovascular naloxone attenuated their antinociception by 70%–80% (compared with a 20%–30% attenuation following intrathecal administration). Therefore, the action of exogenous opioids at specific loci (PAG and RVM) in the descending pain modulatory system is crucial for opioid‐induced analgesia, and it follows that tolerance to their analgesia is due to adaptations in this opiate‐responsive neural circuit.

## 5 MOLECULAR MECHANISMS OF ACUTE OPIOID ANALGESIA

**FIGURE 3:**
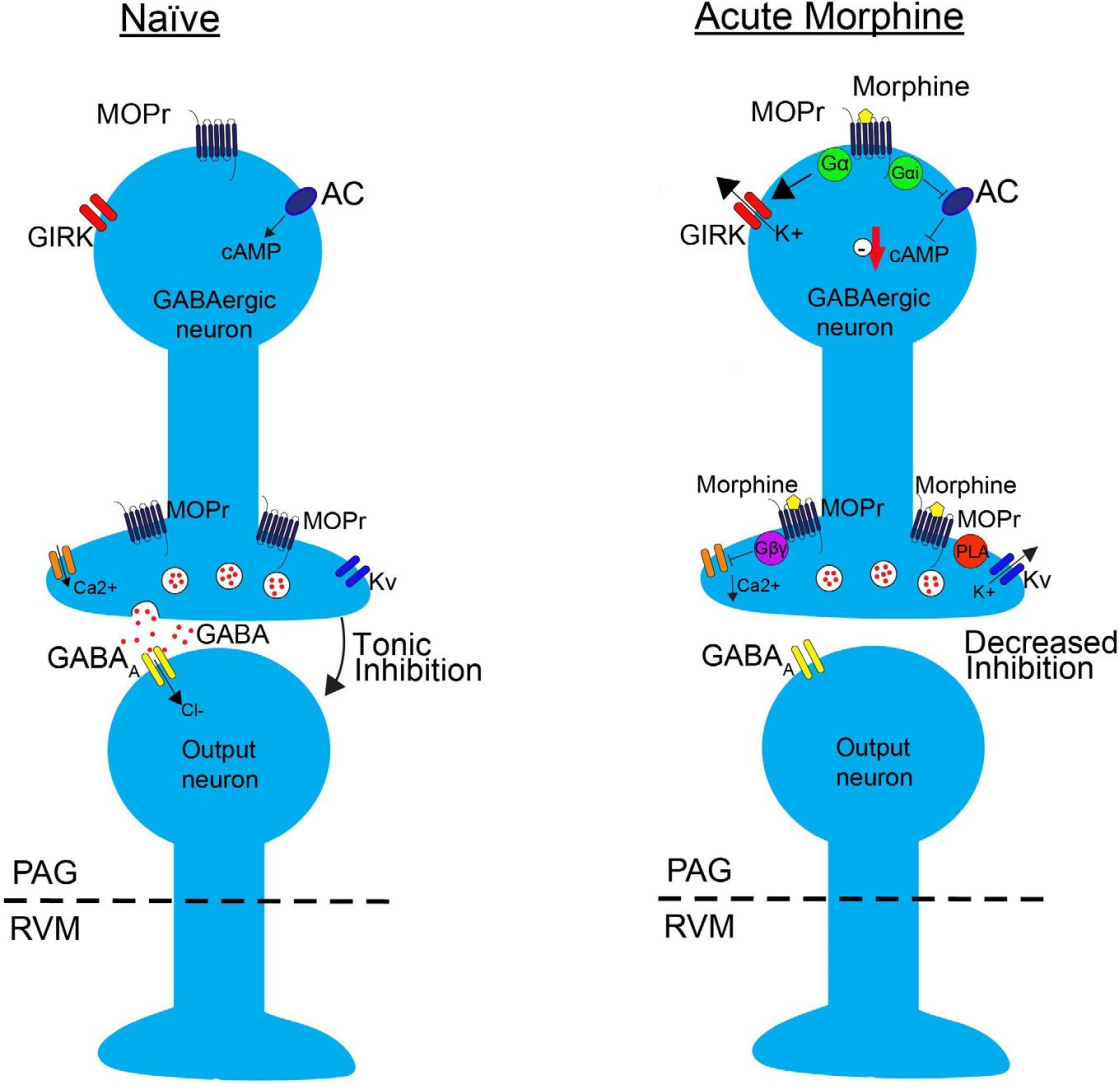
Intracellular signaling in naïve PAG GABAergic interneurons, and following acute morphine administration (reproduced with permission from Lueptow et al. 2018^39^). *In the naïve state*. GABAergic interneurons are tonically active and release the inhibitory neurotransmitter GABA. GABA acts through GABA_A_ receptors on PAG output neurons. GABA_A_ receptors are inhibitory by causing an influx of chloride ions which hyperpolarizes and therefore inhibits the PAG output neurons. *Following acute morphine administration*. MORs initiate a variety of downstream signaling cascades in the interneurons. *Postsynaptically*, MORs activate G‐protein‐coupled inwardly rectifying potassium ion channels (GIRKs), hyperpolarizing the neuron. Both Gα_i_ and Gβγ are important in regulating the activity of GIRK. Gα_i_ directly binds to the GIRK channel, stabilizing it and priming it for Gβγ activation.^40^ *Presynaptically*, MORs inhibit voltage‐gated calcium channels (VGCCs) via the Gβγ subunit. Inhibition by Gβγ is voltage‐dependent and large or repeated depolarizations of the presynaptic terminal could overcome the inhibition.^41^ Gβγ also activates voltage‐gated potassium channels (Kv's) via a mechanism involving phospholipase A (PLA). The overall effect of decrease calcium ion influx and increased potassium ion efflux is hyperpolarization and inhibition of neurotransmitter release, therefore decreasing GABA‐mediated inhibition of output neurons

The PAG is an important region for opioid analgesia. MORs are located on GABAergic interneurons, and are G_i/o_‐coupled GPCRs. Following acute opioid binding, the Gβγ subunit dissociates from the Gα_i_ subunit and both are involved in independent signaling cascades (Figure 3). Gα_i_ inhibits adenylyl cyclase (AC) which decreases cyclic adenosine monophosphate (cAMP) levels resulting in a range of cellular effects, such as decreased protein kinase A (PKA) activation. Interestingly, acute morphine administration appears to rely on the actions of the Gβγ subunit to hyperpolarize GABAergic interneurons (via activation of potassium channels) and directly inhibit voltage‐gated calcium channels (VGCCs) in a membrane‐delimited mechanism. This involves the QXXER motif of the I‐II loop, the intracellular N‐terminus, and the β subunit of the VGCC.^36^, ^37^, ^38^ Inhibition of VGCCs by Gβγ reduces GABA release from the synaptic terminal.^28^ Reduced GABA release disinhibits PAG output to the RVM, increasing spontaneous firing of OFF‐cells responsible for antinociception.

## 6 OPIOID TOLERANCE AND OPIOID‐INDUCED HYPERSENSITIVITY ARE THE RESULTS OF ADAPTIVE CHANGES IN PAIN PROCESSING PATHWAYS

Tolerance and OIH develop with chronic opioid administration. Both are long‐term adaptations that may persist after opioid usage has stopped. The net effect of tolerance and OIH is responsible for the reduction in opioid efficacy observed clinically. The sites and mechanism of tolerance and OIH development must be understood before rational attempts at targeting them can be made.

### 6.1 Sites of tolerance development

Similar levels of analgesic tolerance develop following repeated systemic or local administration of morphine into the vlPAG. Specific activation of vlPAG output neurons by microinjecting bicuculline (a GABA antagonist) and kainite (an excitatory amino acid) did not produce tolerance in rats,^42^ suggesting that repeated activation of output neurons is not sufficient to induce tolerance. Therefore, in the vlPAG, opioids likely induce tolerance via changes in GABAergic interneurons, not the output neurons. This is supported by an observable increase in GABA release in the vlPAG with repeated morphine administration.^43^, ^44^

The development of tolerance within the PAG has downstream effects as activity in RVM OFF‐cells is intrinsically linked to PAG output neuron activity. Acute opioid administration disrupts the activity of ON‐ and OFF‐cells in response to painful stimuli. However, due to tolerance associated with chronic opioid administration, these ON‐ and OFF‐ cells respond as if there was no opioid stimulation.^45^ An increase in ON‐cell activity also occurs following chronic opioid use which appears to counteract opioid analgesia. Nevertheless, this may be associated more with OIH than analgesic tolerance per se. Repeated microinjection of opioids into the RVM results in antinociception (albeit less than that induced by the PAG) but substantially less tolerance,^46^ perhaps because the opioids bypasses the PAG circuitry. Therefore, tolerance develops in numerous sites of the descending system, but the most significant involves GABAergic interneurons in the PAG.

### 6.2 Mechanisms of tolerance in the PAG

When considering potential homeostatic adaptations involved in tolerance, counteracting the direct downstream signaling pathways of MOR activation would lead to decreased responsiveness of the system. Following this, there is strong evidence that “superactivation” of AC occurs in the PAG.

#### 6.2.1 Intracellular changes following chronic opioid use

**FIGURE 4:**
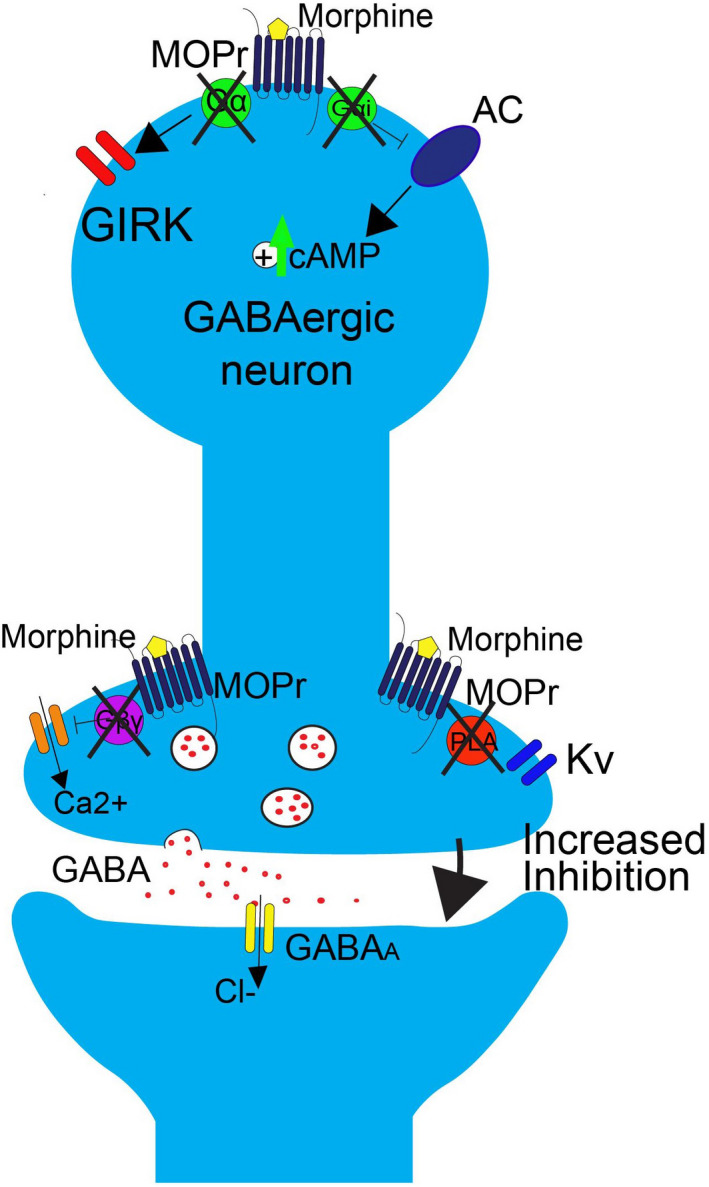
Intracellular adaptations produced by chronic morphine use in GABAergic interneuron in the PAG (reproduced with permission from Lueptow et al.^39^). *Postsynaptically*, there may be some uncoupling between the G proteins and MORs, a process distinct from acute homologous desensitization (Melief et al.^49^; Bruchas et al.^50^), therefore decreased activation of GIRKs. *Presynapticall*y, superactivation of AC results in PKA‐mediated phosphorylation of VGCCs, and therefore increased calcium conductance. The overall effect is increased GABA release and therefore stronger inhibition of PAG output neurons

**FIGURE 5:**
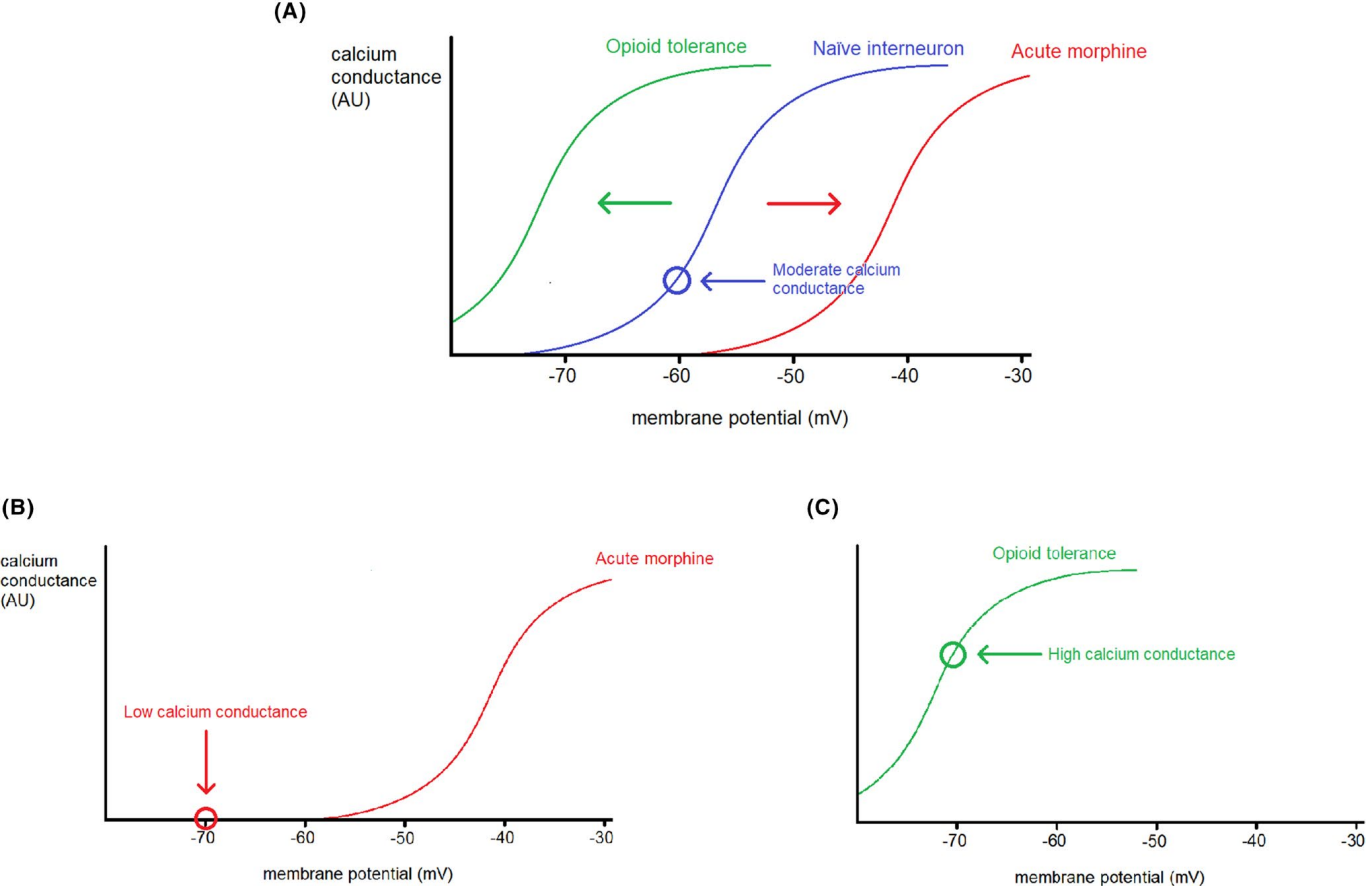
Calcium conductance through VGCCs in naïve, acute, and chronic morphine/withdrawal GABAergic interneurons in the PAG. The average resting membrane potential in PAG interneurons is −60 mV.^51^ A, *the naïve neuron in blue* . a modest level of calcium conductance (arbitrary units, AU) is present. The calcium influx triggers calcium‐dependent exocytosis and neurotransmitter release. Hence naïve PAG interneurons tonically release GABA. B, *acute morphine in red* . following acute activation of MOR, inhibition of AC‐cAMP‐PKA signaling results in dephosphorylation of VGCCs and direct binding of Gβγ causes a right‐ward shift in the activation of VGCCs. Furthermore, the opening of potassium channels by morphine hyperpolarizes the interneurons (approximately −70 mV). The combination of these two factors results in very low conductance through the VGCCs and inhibition of GABA release. C, *chronic morphine in green* . superactivation of AC causes overactivation of PKA that can phosphorylate VGCCs. Phosphorylation by PKA increases the probability of channel opening, allowing for calcium influx even in hyperpolarized neurons^52^

Chronic morphine use produces adaptations contributing to opioid‐tolerance within MORs’ downstream signaling pathways (Figure 4). For example, whereas acute morphine inhibits AC, reducing cAMP levels, chronic morphine upregulates cAMP via “superactivation” of AC. Compensatory activation of AC increases cAMP concentrations in the neurons, which has a variety of cellular effects. Of significance, phosphorylation of VGCCs by PKA causes a leftward shift in their activation curve (Figure 5). Heightened calcium conductance increases inhibition of PAG output neurons via GABA_A_ receptor activation. Supporting this, potassium channel blockers abolished acute opioid presynaptic inhibition of GABA release in the PAG but had no effect on the increased inhibitory synaptic currents observed with chronic opioids, whereas PKA inhibitors did block this.^47^ Therefore, acute opioid antinociception involves Gβγ‐dependent pathways, but signaling in presynaptic terminals becomes increasingly dependent on AC after continuous administration of morphine. This switch to dependence on the AC‐cAMP‐PKA pathway is not specific to the PAG and in other regions contributes to withdrawal behaviors.^48^

#### 6.2.2 Inhibition of adenylyl cyclase as a method to reduce tolerance

AC seems to be a promising target to attenuate tolerance without effecting acute opioid efficacy, based on animal studies. A recent experiment demonstrated that repeated activation of AC in the vlPAG by forskolin mimics morphine tolerance, and blocking AC with an inhibitor reverses morphine tolerance.^53^ In theory, inhibition of AC would prevent the left‐ward shift of VGCCs caused by AC superactivation without effecting antinociception via Gβγ‐dependent signaling pathways. Furthermore, AC inhibition may provide the added benefit of reversing many of the withdrawal symptoms associated with cessation of chronic opioid use.^53^ Thus, AC may be a good target to reduce tolerance development. A major limitation of this method is the inability to specifically target AC inhibitors to important brain regions such as the PAG in humans. Systemic administration would result in too many side‐effects and would interfere with many other drugs on the market. Microinjections are used in animal studies, but this is not viable in humans requiring long‐term opioid use. Targeted drug delivery systems are currently not reliable, but the significant amount of modern research into new delivery vehicles for active targeting makes the potential use of AC inhibitors a very real possibility in the future.^54^ Alternative methods to reduce AC levels also exist, for example, targeting other G_i/o_‐receptors expressed on the same MOR‐responsive GABAergic interneurons. GABA_B_ receptors are a potential target; they are G_i/o_‐coupled and are expressed in almost all PAG neurons.^55^ Likewise, cannabinoid receptors (CB_1_ receptors) are G_i/o_‐coupled and are expressed in PAG GABAergic interneurons.^56^

#### 6.2.3 Coadministration of cannabinoids to increase vlPAG output

Another potential method of improving clinical opioid efficacy and reducing tolerance development is through drugs that activate PAG output neurons via distinct mechanisms from opioids. As discussed, this could be achieved by targeting other G_i/o_‐coupled receptors on MOR‐expressing GABAergic interneurons. Manipulating the endocannabinoid system appears an ideal candidate. CB_1_ receptors are expressed in many of the same regions as MORs, and there is strong evidence to show that CB_1_ receptor agonists are capable of producing analgesia.^57^ Like the opioid system, endocannabinoids are released as a neurotransmitter from specific neurons under certain stressful conditions.^58^ Targeting this system with exogenous cannabinoids overcomes the requirement for these specific conditions.

Like opioid receptors, CB_1_ receptors are G_i/o_‐coupled GPCRs. They also presynaptically inhibit GABAergic synaptic transmission to disinhibit PAG antinociceptive output neurons. However, while MORs directly inhibit tonically active GABAergic interneurons in the PAG,^59^ cannabinoid‐induced increases in PAG output appears to be dependent on metabotropic glutamate receptor 5 (mGlu5R) activity. A selective mGlu5R antagonist microinjected into the PAG completely blocked the effect of exogenous cannabinoid agonists on PAG cell activity.^60^ This is consistent with previous findings that glutamate can produce antinociception in the PAG via postsynaptic mGlu5Rs on output neurons.^61^ The source of this glutamate may be from excitatory glutamatergic interneurons in the PAG. Activation of the second population of GABAergic interneurons expressing CB_1_ receptors (distinct from MOR containing GABAergic interneurons in the PAG) disinhibits the glutamatergic interneurons, thus increasing the activity of the PAG output neurons. Supporting this, a recent experiment found chemogenetic inhibition of CB_1_ expressing GABAergic neurons in the vlPAG activated glutamatergic interneurons in the vlPAG, producing antinociception.^62^ Confirmation of this mechanism requires receptor expression studies to identify if CB_1_ receptors and MORs are co‐expressed in PAG GABAergic interneurons, or if they are found in distinct populations.

Cannabinoids also act directly in the RVM, shifting the balance between ON‐ and OFF‐cells in the RVM in the direction of the antinociceptive OFF‐cells. Systemic administration of cannabinoids inhibits RVM ON‐cell activity and increases OFF‐cell activity.^63^ This mechanism appears to be via direct action in the RVM. CB1 receptor agonists increase the spontaneous activity of OFF‐cells following microinjection into the RVM.^64^ This is distinct from the action of MOR agonists in the RVM. Microinjection of morphine to the RVM only depressed ON‐cell activity, without effecting OFF‐cell firing,^34^ suggesting only ON‐cells expressed MORs.^33^ As such, even though the end result of CB1 agonists on the PAG‐RVM system is similar to that of opioids (i.e., increased activity of OFF‐cells and decreased activity of ON‐cells), cannabinoids may offer complementary analgesia when co‐administered with opioids by shifting the balance between the state of ON‐ and OFF‐cell firing at both the level of the PAG and RVM, through mechanisms independent of MORs and bypassing opioid tolerance mechanisms in the PAG.^33^

#### 6.2.4 Cannabinoids may improve the efficacy of opioids in certain neuropathic pain conditions

**FIGURE 6:**
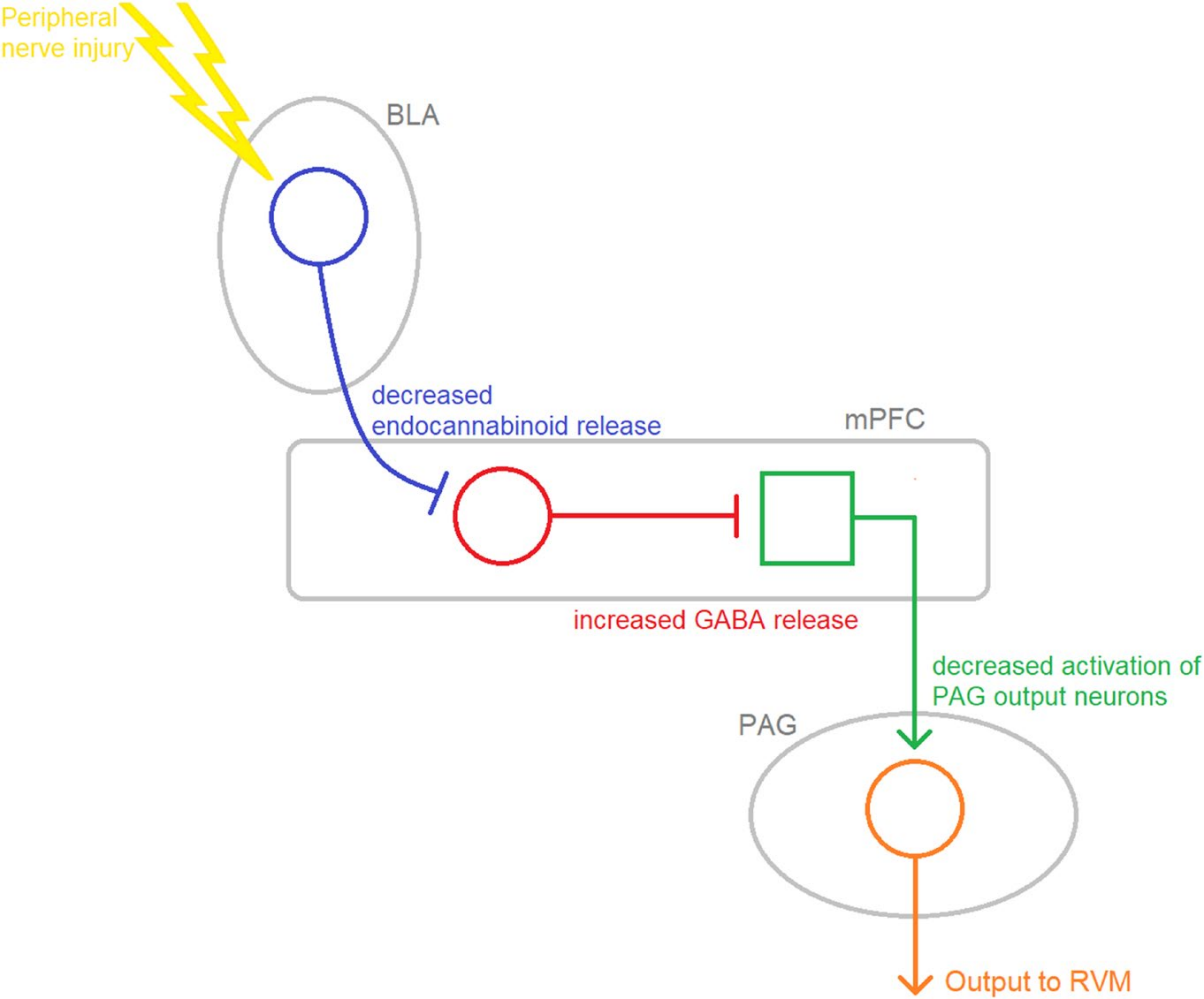
Linear circuit involved in the development of neuropathic pain (as described by Huang et al.^65^). Peripheral nerve injury augments basolateral amygdala (BLA) inputs onto GABAergic interneurons located in the medial prefrontal cortex (mPFC). This augmentation is the result of weakened endocannabinoid signaling. Decreased CB_1_ receptor density was observed in BLA‐originating presynaptic terminals. Increased activity in mPFC inhibitory interneurons leads to an overall inhibition of excitatory pyramidal cell output toward vlPAG output neurons. The net effect is decreased descending inhibition via the RVM to the spinal cord

A linear circuit involved in the development of neuropathic pain has recently been described by Huang et al.^65^ (Figure 6).

This linear circuit interacts with the opioid‐sensitive descending system. PAG projection neurons to the RVM receive excitatory input from the medial prefrontal cortex (mPFC) and inhibitory input from GABAergic interneurons in the PAG. The balance of these two inputs dictates the level of descending pain modulation. Opioid action in the PAG disinhibits output neurons. However, in some cases of neuropathic pain, decreased excitatory connections between the mPFC and vlPAG means the action of opioids alone on PAG interneurons cannot cause substantial activation of the descending pain system. Essentially, the neuropathic condition itself has reduced the effectiveness of the PAG‐RVM‐DH antinociceptive system. This model suggested by Huang et al. outlines a potential synergistic role of cannabinoids and opioids in treating patients suffering from certain neuropathic conditions who do not receive adequate analgesia from opioids alone, by allowing for a greater level of descending pain inhibition. However, the current research in this area does not go beyond in‐vitro studies and, moreover, specific neuropathic conditions which might be modulated by this circuit are unknown. Thus, more active research into this area is needed.

A recent meta‐analysis found a significant reduction in neuropathic pain in patients receiving cannabinoid treatment.^66^ Additionally, the use of cannabis as an adjunct to opioids may provide greater cumulative relief of pain and allow for a reduction in opioid dose, decreasing opioid‐related side‐effects.^67^ Further clinical trials are required to fully profile the side‐effects of cannabinoid treatment and identify the types of pain they may be useful for. The recent legalization of cannabis in many countries and widespread availability make targeting the endocannabinoid system a compelling avenue to pursue.

### 6.3 Sites of OIH development

Hyperalgesia has been reported in several laboratories following systemic opioid administration.^68^, ^69^ Clinical data support this experimental evidence, for example, in patients detoxing from high opioid doses.^70^ A prospective trial in which participants were given morphine for lower back pain also demonstrated measurable hyperalgesia within one month.^71^ Therefore, OIH is a well‐documented phenomenon, distinct from opioid tolerance, and unrelated to changes in underlying pain pathology. Often, the development of OIH is only observed following withdrawal from opioids, as high opioid doses can mask the hyperalgesia.

The development of OIH involves a variety of independent adaptive changes in the opioid‐responsive pain processing pathways. Chronic morphine administration increases the number of active ON‐cells in the RVM, likely causing pain hypersensitivity.^72^ Furthermore, OIH also incorporates sensitization of the ascending pro‐nociceptive pathway; for example, increased activity of primary afferents within dorsal root ganglia and sensitization of spinal neurons in the DH.^73^, ^74^ Many of the mechanisms leading to OIH appear to alter synaptic plasticity between neurons, leading to a more pronociceptive state in the body.

### 6.4 Mechanisms of OIH development in the RVM

Descending facilitation from the RVM to the spinal cord is important for the manifestation of OIH. Continuous morphine administration results in a hypersensitive state which can be reversed by lidocaine injection into the RVM.^75^ The mechanism of OIH in RVM appears to involve a range of pronociceptive neuropeptides which act through similar mechanisms.

#### 6.4.1 Cholecystokinin is a pronociceptive peptide that drives descending facilitation from the RVM

Microdialysis of rat RVMs following continuous systemic morphine showed a fivefold increase in cholecystokinin (CCK) levels compared with controls, suggesting that CCK release is one of the long‐term signaling outcomes of morphine administration.^76^ Furthermore, activation of CCK receptors in the RVM promoted mechanical and thermal hypersensitivity,^76^ whereas CCK antiserum or administration of receptor antagonists prevented this.^77^ Therefore, activation of the CCK system by opioids may play a role in the development of OIH, and animal studies have shown the potential of CCK antagonists in attenuating this phenomenon. The source of CCK output to the RVM appears to involve inputs to the RVM, rather than a direct release from RVM interneurons, as local administration of morphine into the RVM did not cause CCK release.^76^

CCK in the RVM modulates ON‐ and OFF‐cell firing. CCK receptors are a group of G_i/o_‐coupled GPCRs, and activation hyperpolarizes neurons, reducing neurotransmitter release.^78^ Activation of CCK receptors in the RVM prevents the morphine‐induced increase in OFF‐cells activity.^79^ A more recent study found CCK microinjection into the RVM activated ON‐cells, perhaps mediating descending pain facilitation.^80^ Activation of ON‐cells may be mediated through an excitatory G_q_‐coupled CCK receptor. The reliability of CCK‐mediated activation of ON‐cell firing has been questioned as many studies have failed to repeat this finding. One hypothesis is that higher doses of CCK activate ON‐cells; Heinricher & Neubert (2004) used a concentration 3x higher than Heinricher et al. (2001).

Preliminary human evidence suggests that CCK is a viable target to improve opioids, with increased opioid efficacy being recognized as a novel side effect of proglumide (a non‐selective CCK antagonist) in human volunteers.^81^ However, little progress has been made over the last 30 years in regard to clinical trials of CCK antagonists for this purpose. This lack of progress may be the consequence of insignificant results in humans due to redundancy within the “anti‐opioid” system. Other neuropeptides, such as neuropeptide FF (NPFF), are also able to induce OIH and targeting only one anti‐opioid may not significantly impact hypersensitivity in non‐experimental models.^82^ Rather than targeting the neuropeptides directly, it may be useful to identify the MOR signaling cascades responsible for activating the anti‐opioid systems with the hopes of discovering a common targetable pathway.

### 6.5 OIH in the spinal cord and peripheral nervous system

Numerous mechanisms for OIH have also been identified in the spinal cord and peripheral nervous system including sensitization of primary afferents and enhanced glutamate release from these afferents, hyperexcitability of second‐order neurons, and increased descending facilitation.^83^

#### 6.5.1 The role of spinal neuroinflammatory cells in OIH

Opioids can trigger pro‐inflammatory cascades in astrocytes and microglia in the spinal cord, where they may contribute to OIH. Following opioid binding to the MOR, various intracellular signal pathways are activated leading to pro‐inflammatory cytokine release and a shift in chloride activity from inhibitory to excitatory.^84^ These adaptive changes mimic the development of hyperalgesia in various inflammatory and neuropathic pain conditions.^85^ Acute opioid administration is not sufficient, and chronic opioid use is required, for the activation of astrocytes and microglia.^86^, ^87^

The contribution of neuroinflammatory cells has been demonstrated using inhibitors of glial cell activity ‐ either glial cell blockers or antagonists of the mediator's released (e.g., proinflammatory cytokine antagonists). Furthermore, cytokines and chemokines released may contribute to OIH. Chronic morphine upregulated C‐X‐C chemokine receptor type 4 (CXCR4) in nociceptors, and blockade with antagonists reversed OIH in rats.^88^ These neuroinflammatory molecules create ideal targets as many drugs targeting them are already available for use in the clinic.

#### 6.5.2 Microglia activation shifts the neuronal anion gradient making previously inhibitory synapses excitatory

**FIGURE 7:**
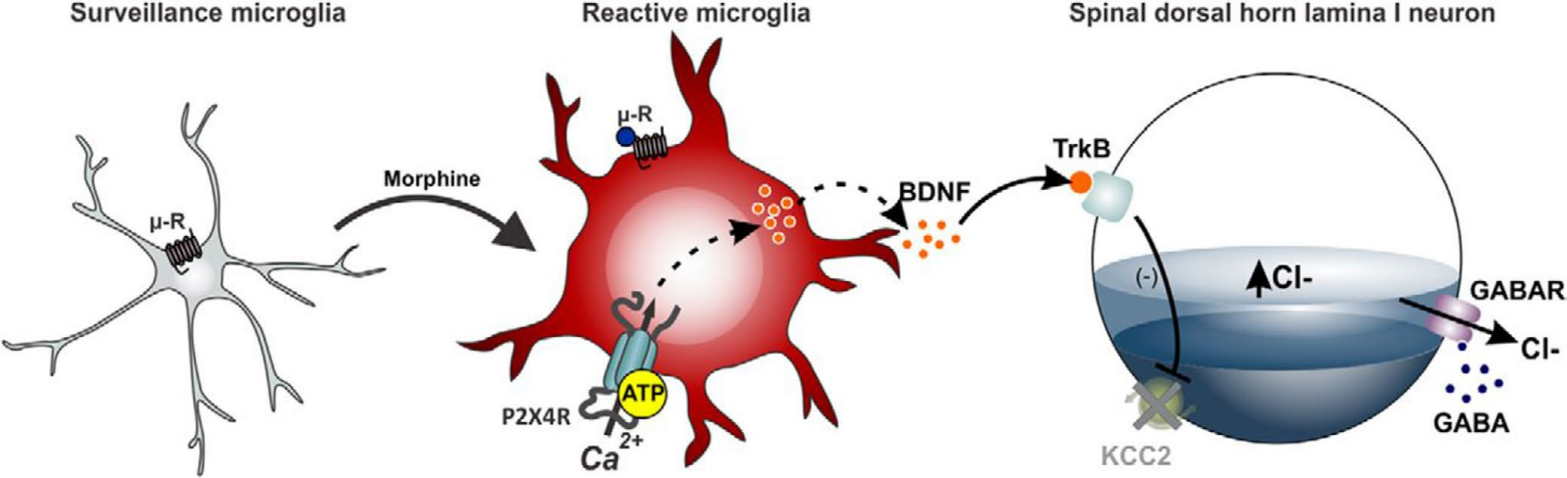
The P2X4‐BDNF‐KCC2 pathway involved in OIH (reproduced with permission from Trang et al. 2015). The binding of morphine (or other MOR agonists) to MORs on spinal microglia activates pro‐inflammatory cascades in the microglia. Microglia activation induces P2X4 receptor upregulation, and morphine causes the release of BDNF through ATP‐mediated stimulation of P2X4 receptors. BDNF acts through TrkB on dorsal horn lamina I neurons to downregulate the expression of KCC2. This disrupts chloride homeostasis in the DH by preventing chloride efflux via KCC2. The increased intracellular chloride concentration in lamina I neurons shifts GABA receptor activation from inhibitory to excitatory^90^

Brain‐derived neurotrophic factor (BDNF) is released from microglia following opioid binding and activates tropomyosin receptor kinase B (TrkB) on DH neurons, inverting the polarity of current activated by GABA.^89^, ^90^ Normally, influx of chloride through GABA receptors is inhibitory. However, morphine disrupts neuronal chloride homeostasis and instead, chloride exits through activated GABA receptors to cause depolarization. Gene‐targeted mice in which BDNF was deleted from microglia did not develop hyperalgesia to morphine, but still developed tolerance, dissociating the two phenomena.^90^ The mechanism of microglia‐mediated OIH involves a pathway beginning with P2X‐purinoceptor 4 (P2X4) receptors in microglia, and ends with downregulation of the potassium‐chloride co‐transporter (KCC2) in DH lamina I neurons (Figure 7). The loss of chloride homeostasis may also have implications on the outcome of the descending pain system. A large proportion of OFF‐cells activated in the RVM are GABAergic and act directly at synapses of ascending neurons in the DH. As such, what little descending inhibition from the RVM remains due to tolerance, is not as effective.^32^

Understanding the P2X4‐BDNF‐KCC2 pathway creates numerous novel pharmacological targets to improve opioids by attenuating OIH. Importantly, the goal is to restore Cl^−^ extrusion to allow GABA receptors to function normally. Furthermore, BDNF‐TrkB signaling also appears important for the development of inflammatory and neuropathic pain conditions, so targeting this pathway may provide a dual role by acting as an opioid adjuvant to reduce OIH and improving the chronic pain condition itself.^91^, ^92^

#### 6.5.3 Activation of the central glutaminergic system through NMDA receptors plays a crucial role in OIH

The glutamatergic system in the DH is crucial for OIH by increasing the strength of synaptic transmission between neurons. Glutamate N‐methyl‐D‐aspartate receptors (NMDARs) are located presynaptically on primary afferents and postsynaptically on spinal DH neurons, so are well placed to induce long‐term potentiation (LTP) in the ascending pain processing pathway.^93^ LTP produces a long‐lasting increase in signal transmission between two neurons.^94^ Opioids can activate this central glutaminergic system via sustained NMDAR activity, inducing LTP and sensitizing DH neurons.^95^ Supporting this, LTP has been shown to occur between primary afferent C‐fibers and neurons from the superficial layers of the DH.^96^

Administration of ketamine (a NMDAR antagonist) diminishes OIH in both rats and mice,^68^, ^97^ supporting the role of glutamate in OIH. Electrophysiology recordings showed significantly increased amplitude and frequency of excitatory postsynaptic currents evoked from primary afferents following chronic morphine, and this was attenuated by blocking protein kinase C (PKC) or with NMDAR antagonism.^98^ They hypothesized that chronic morphine administration induced PKC‐mediated phosphorylation of NMDARs. Phosphorylation of NMDARs overcomes their characteristic Mg^2+^ block, surpassing the need for depolarization, to allow Ca^2+^ conductance. PKC also promotes NMDAR trafficking to the plasma membrane.^99^ Increased Ca^2+^ flux through NMDAR causes activation of Ca^2+^/calmodulin‐dependent protein kinase II, PKA and neuronal nitric oxide synthase (nNOS), and further activation of PKC. While the effects of these specifically for analgesia are not clear, their effects in other neuronal circuits may provide an insight into their functions. For example, activation of nNOS increases the synthesis of nitric oxide (NO) from L‐arginine. NO in presynaptic terminals increases glutamate neurotransmitter release.^100^ This supports a potential role of PKC inhibitors and NMDAR antagonists in reducing OIH. Furthermore, chronic morphine decreases the glutamate transporters glutamate aspartate transporter EAAT1 (GLAST‐1) and glutamate transporter EAAT2 (GLT‐1) activity in neurons, sustaining the increased synaptic glutamate concentration and further enhancing glutamate signaling in the DH.^101^

While NMDAR antagonist have been shown effective at increasing opioid potency in animal models, they have not passed clinical trials for this purpose due to major side‐effects such as hallucinations and drowsiness,^102^ the result of excessive NMDAR blockage. Hypothetically, an NMDAR antagonist that maintains activity at normal physiological levels would prevent side effect while also attenuating OIH. The endocannabinoid system has recently emerged as an endogenous regulator of NMDAR activity and studies have observed CB_1_ receptors directly interacting with NMDARs, reducing their activity.^103^ Furthermore, CB_1_ receptors are expressed in the DH.^104^ Therefore, cannabinoids may also be beneficial for reducing OIH. Alternatively, EAAT2 activators may have a role in reversing OIH. The β‐lactam antibiotic ceftriaxone upregulates spinal EAAT2 in an animal model of multiple sclerosis, and reverses the associated hyperalgesia.^105^ Further experiments to confirm these findings in animal models of OIH are required. Nonetheless, EAAT2 also appears a promising target for therapeutic intervention.

## 7 RECEPTOR AND CELLULAR SIGNALING OF TOLERANCE/OPIOID‐INDUCED HYPERSENSITVITY

**FIGURE 8:**
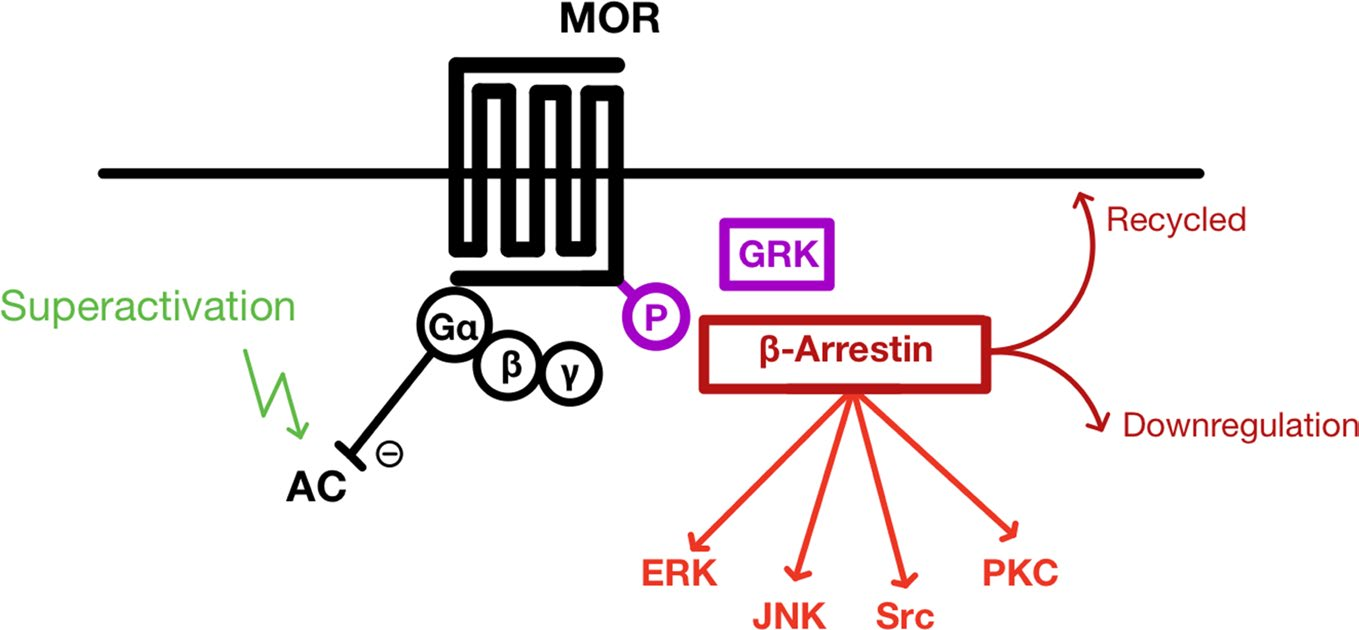
The various signaling outcomes following MOR activation. G‐protein‐dependent signaling following MOR activation involves the G‐protein subunits pre‐bound to the receptor, in the case of MOR, this is usually the Gα_i_ and Gβγ subunits. Acutely, MOR activation inhibits adenylyl cyclase (AC) through Gα_i_, but chronic MOR activation appears to over‐activate AC. Desensitization, internalization, recycling, and downregulation involve agonist‐induced receptor phosphorylation, for example, through GRK. β‐arrestin is recruited by phosphorylated residues of MOR. β‐arrestin binding can trigger clathrin‐mediated internalization of the receptor. MORs in endosomes can either be recycled to the plasma membrane or broken down (downregulation). β‐arrestin can act as a scaffold to activate G‐protein‐independent signaling cascades (in red), for example, extracellular signal‐regulated kinases (ERKs), c‐Jun N‐terminal kinases (JNKs), Src, and PKC^106^

MOR activation leads to a variety of signaling cascades and receptor adaptations and some may be good pharmacological targets (Figure 8, Table 1 and 2) to improve the opioid side‐effect profile and attenuate the development of tolerance and OIH.

**TABLE 1:** Summary of the proteins involved in G‐protein‐dependent signaling and desensitization, internalization and downregulation, and their suitability as pharmacological targets to improve opioids

Protein implicated	Role in analgesia	Role in tolerance/OIH	Suitable pharmacological target?
Adenylyl cyclase (AC)	Refer to Section 6.2.2		
Gi/Gs	Naïve and acute MORs are coupled to Gi. The Gi signaling cascade is essential for opioid‐induced analgesia.	Switch of the MOR‐coupled G‐protein from Gi to Gs in some spinal dorsal horn neurons, reversing the effect of opioids (tolerance) and inducing hyperalgesia^107^	Targeting Gs itself will be associated with too many side effects not related to MOR. Enhanced activity of the pronociceptive adrenomedullin (AM) can induce the switch from Gi to Gs‐coupled MORs^108^ and this represents a more specific target to attenuate tolerance.
GRK/β‐arrestin	N/A	In theory, desensitization of MOR (uncoupling of G‐proteins to the receptor) and downregulation in key regions associated with analgesia could result in tolerance.	Desensitization is an acute phenomenon and occurs as part of the normal physiological response to GPCR activation. It is unlikely to contribute to the level of chronic opioid tolerance observed in the clinic. Also, downregulation of MOR has not been observed in regions important for analgesia. Morphine tolerance is associated with a decrease in opioid‐mediated inhibition of GABA release that is not a result of MOR desensitization.^109^

**TABLE 2:** Summary of the proteins involved in G‐protein‐independent signaling their suitability as pharmacological targets to improve opioids

Protein implicated	Role in analgesia	Role in tolerance/OIH	Suitable pharmacological target?
β‐arrestin	N/A	N/A	Provides a scaffolding role and is not a signaling molecule in its own right, therefore unlikely to be a good pharmacological target.^110^
Mitogen‐activated protein kinase pathways (ERK and JNK)	Contribution toward antinociception appears to my both agonist and location dependent.^111^	Similar to their role in antinociception, ERK and JNK’s role in tolerance is agonist and cell location dependent.^111^, ^112^	Differential functions of ERK and JNK make this a poor pharmacological target based on our current molecular understanding of their signaling pathways.
Src	Src activation appears to have no effect on antinociceptive effect^113^	The MOR‐B‐arrestin‐Src complex can phosphorylate AC isoforms (contributing to AC overactivation) and other proteins (e.g., MAPK, GRK2/3) implicated in tolerance and OIH^114^, ^115^	Src kinase inhibition attenuates morphine tolerance.^113^ Inhibitors of Src (e.g., Dasatinib) are already used clinically for leukemia, so their safety in humans is already established.
PKC	N/A	PKC has been implicated in acute desensitization of MOR in response to specific opioid agonists, analogous to GRK^116^; PKC activation by MORs may sensitize NMDAR receptors co‐expressed on postsynaptic DH neurons, increasing NMDAR activation.^117^	Acute desensitization does not appear significant toward chronic opioid tolerance. While there may be some potential in directly targeting PKC to reduce activation of downstream effectors (e.g., NMDAR), no PKC inhibitor has been approved for clinical use.^118^ Targeting the downstream effectors directly may be more successful

A remaining mystery in the development of analgesic tolerance and OIH is the link between acute opioid signaling outcomes and the establishment of neuronal adaptations observed chronically. The development of many novel experimental drugs relies heavily on observed empirical data, with little mechanistic insight into why they work. For example, the role of B‐arrestin‐dependent signaling pathways is not clear (Table 2). Bohn et al. found attenuated analgesic tolerance to morphine in β‐arrestin2 knockout mice, but similar knockouts did not alter tolerance to fentanyl.^119^ Similar differences have been observed for the respiratory depression and GI symptoms in arrestin knockout mice.^120^, ^121^ The mechanisms underlying these discrepancies are still not clear, but it is becoming increasingly apparent that attributing the adverse effects of MOR ligands to arrestin signaling may represent an oversimplification of the pathways.^122^, ^123^ Instead, recent evidence supports a role of G‐proteins and arrestin signaling pathways in both the antinociceptive and the major side effects associated with MORs.

### 7.1 Development of novel biased opioid receptor ligands to improve opioids

The two most prescribed “strong opioids” in the UK are morphine and fentanyl. Fentanyl was synthetically developed with a higher affinity for MOR, with the intention that it would make for an improved opioid. However, clinical evidence has not supported this, suggesting similar analgesic efficacies and tolerance development between the two opioids. More recently, a range of experimental G‐protein/βarr2 biased MOR agonists has been developed and tested in mouse models, with the hope that there may be an optimum ratio of G‐protein:B‐arrestin signaling that would result in an “improved” opioid.^124^ Bias is related to different conformational states of the receptor, triggered by structural variations of the ligand and the transducers present.^125^ Agonists highly biased toward G‐proteins with poor recruitment of B‐arrestin2 appear to have more favorable properties, including higher antinociceptive potential and attenuated development of tolerance.

One such MOR G‐protein‐biased ligand, TRV130, was shown to cause less GI dysfunction and respiratory depression than morphine at equianalgesic doses in rats.^121^ Preclinical data of TRV130 suggested it may be a safer and less tolerant prone opioid for treating chronic pain. Despite promising preclinical data, TRV130 completed phase III clinical trials with underwhelming results, demonstrating only a trend toward reduced side effects but no significant difference compared with morphine.^126^ Therefore, optimizing bias factor alone may not be enough to improve opioids.

### 7.2 Peripheral/central site of action alters the side‐effect profile of opioids

The distribution of opioids in the body following systemic administration impacts its outcomes. Peripherally restricted opioids that have a poor affinity for crossing the blood‐brain barrier would be expected to develop less analgesic tolerance and respiratory depression as these are largely centrally mediated side‐effects. However, peripherally restricted opioids do not appear to offer the same level of analgesic coverage for painful conditions as centrally acting opioids. Therefore, peripherally acting opioids may only be useful in pain‐causing conditions lacking central sensitization.^127^ Herkinorin, a novel MOR selective G‐protein‐biased ligand, has a reduced tolerance profile and remains efficacious in rats made tolerant to chronic morphine, but its effects are peripherally restricted to the site of injection.^128^

### 7.3 Opioid kinetics

Bias is not the only factor that can alter the signaling outcome of a GPCR ligand. The kinetics of the drug, for example, its residual time (the time the drugs spends bound to the receptor), also appear important; buprenorphine and TRV130 both have similar G/Barr‐biases, but buprenorphine dissociates significantly slower from MORs and has an 18x higher residence time compared to TRV130.^129^ This difference could potentially lead to significantly different clinical effects, such as the lower respiratory depression reported with buprenorphine.^130^ The effect of residual time on opioid efficacy has not yet been researched but this may be another parameter that requires fine optimization.

Taken together, optimizing the bias, the residual time and the degree of central/peripheral action may be the next step in developing the “perfect” opioid agonist. The development of novel opioids, such as endorphin derivatives that induce less tolerance with no significant OIH or glial activation,^131^ and biased‐MOR agonists, indicates that opioids possessing good analgesic properties with reduced OIH and tolerance is possible.

## 8 CONCLUSION

Chronic pain is not merely prolonged activation of normal pain pathways, but instead reflects plasticity in both peripheral and central neuronal circuits. Likewise, long‐term opioid signaling is not simply inhibition of afferent pain signaling but involves adaptive changes in all the major pain processing pathways, some of which resemble the changes that occur in chronic pain.

As a result of the severe side‐effect associated with opioids, much of pain research over the last decades has attempted to replace opioids, mostly with little fruition. Perhaps it is time to take a step back and look at improving opioids, harnessing their undeniable analgesic efficacy of the opioid‐responsive system. A variety of novel targets have been discussed that, when co‐administered with an opioid, may allow suffers of chronic pain to achieve effective long‐term pain relief. Furthermore, the development of new opioid agonists with more desirable properties is also a possibility. Tolerance and OIH are just two of the factors that plague opioid usage. Research must also be done into minimizing opioid addiction and physical dependence. Addiction involves of elements of biology but also behavioral, societal, and other social factors such as education, so any improved opioid paradigm must involve more than just pharmacological improvements.^132^ For instance, a greater involvement of healthcare professionals, including psychiatrists, may be beneficial in long‐term opioid users.^133^ By championing multiple avenues of investigation such as improving opioids by minimizing OIH and tolerance as discussed in this review, improving opioid addiction and physical dependence, and continuing work on non‐opioid alternatives, the likelihood of improving analgesic treatment becomes ever greater.

MOR variants within individuals result in varying responses to opioids, and differing mechanisms and propensities to tolerance and OIH.^134^ As such, it is likely that improving opioid treatment will vary on a case by case basis. In the future, trialing each patient on a specific “improved” opioid agonist with an adjuvant (e.g., AC inhibitors, cannabinoids, CCK receptor antagonists, or NMDAR antagonists), and refining this combination over time, will likely result in the best combination for the individual.

## CONFLICT OF INTEREST

The authors declare no conflicts of interest.

## ETHICS COMMITTEE APPROVAL

No ethics committee approval was sought.

## Data Availability

Data sharing not applicable to this article as no datasets were generated or analyzed during this paper.
